# Spin-current nano-oscillator based on nonlocal spin injection

**DOI:** 10.1038/srep08578

**Published:** 2015-02-26

**Authors:** V. E. Demidov, S. Urazhdin, A. Zholud, A. V. Sadovnikov, A. N. Slavin, S. O. Demokritov

**Affiliations:** 1 Department of Physics and Center for Nonlinear Science, University of Muenster, 48149 Muenster, Germany; 2 Department of Physics, Emory University, Atlanta, GA, USA; 3 Saratov State University, Saratov 410012, Russia; 4 Department of Physics, Oakland University, Rochester, MI, USA; 5 Institute of Metal Physics, Ural Division of RAS, Yekaterinburg 620041, Russia

## Abstract

Nonlocal spin injection has been recognized as an efficient mechanism for creation of pure spin currents not tied to the electrical charge transfer. Here we demonstrate experimentally that it can induce coherent magnetization dynamics, which can be utilized for the implementation of novel microwave nano-sources for spintronic and magnonic applications. We show that such sources exhibit a small oscillation linewidth and are tunable over a wide frequency range by the static magnetic field. Spatially resolved measurements of the dynamical magnetization indicate a relatively large oscillation area, resulting in a high stability of the oscillation with respect to thermal fluctuations. We propose a simple quasilinear dynamical model that reproduces well the oscillation characteristics.

Injection of pure spin currents has recently become recognized as an efficient route for the control of the dynamic magnetic damping^1^,^2^,^3^,^4^,^5^,^6^,^7^,^8^,^9^,^10^ and excitation of coherent magnetization auto-oscillations in magnetic nanostructures^11^,^12^,^13^,^14^,^15^,^16^. In contrast to the devices operated by spin-polarized electrical currents^17^, spintronic devices driven by pure spin currents are expected to be less affected by the heating and electromigration effects caused by the charge flow through the active magnetic layer. Separation of charge and spin flows also eliminates a number of constraints on the geometry and thus on the magnetic characteristics of devices.

Pure spin currents generated by the Rashba or the spin Hall effect (SHE) in non-magnetic materials with strong spin-orbital coupling^18^,^19^,^20^,^21^ have been already demonstrated to enable efficient control of the magnetization^1^,^2^,^3^,^4^,^5^,^6^,^7^,^8^,^9^,^10^,^11^,^12^,^13^,^14^,^15^,^16^,^21^. However, the same spin-orbit effects that produce spin currents also adversely affect the dynamical characteristics of the adjacent ferromagnets^1^,^2^,^5^.

Pure spin currents can be also generated in the nonlocal spin valve structure^22^,^23^, where charge and spin currents are separated by providing an additional current path that bypasses the active magnetic layer. This approach eliminates the detrimental effects of layers with strong spin-orbital coupling. Moreover, physical separation of the electric- and spin-current paths in nonlocal spin valves can reduce the effect of the current-induced Oersted field on the active ferromagnet, resulting in simpler dynamical behaviors that can provide a significant insight into the mechanisms of auto-oscillation in nanomagnetic systems. Nonlocal spin injection has been intensely studied in recent years. It was found to enable magnetization switching^24^,^25^ and modulation of the dynamic magnetic damping^26^,^27^. However, excitation of coherent magnetization dynamics in nonlocal devices has not been achieved so far.

Here we report an experimental observation of magnetization auto-oscillations due to the injection of pure spin current in a nonlocal spin valve structure. We demonstrate that this mechanism enables generation of coherent magnetization precession with the frequency tunable in the range 6–12 GHz and the linewidth below 20 MHz. Spatially resolved measurements show that, similarly to the conventional spin-torque and spin-Hall nano-oscillators with in-plane magnetization, the auto-oscillation mode excited by the pure spin current in the nonlocal spin valve devices is a localized mode with the frequency below the spectrum of propagating spin waves. However, in contrast to most of the previously studied devices, the localization area is relatively large (about 300 nm), suggesting that the spatial extent of the auto-oscillation mode is not significantly affected by the nonlinear self-localization effects, but is rather determined by the spin-diffusion length in the current-carrying electrode. This conclusion is reinforced by the results of micromagnetic simulations based on a simple linear normal-mode model that provide a good agreement with the experimental data.

## Results

### Test devices

**Figure 1:**
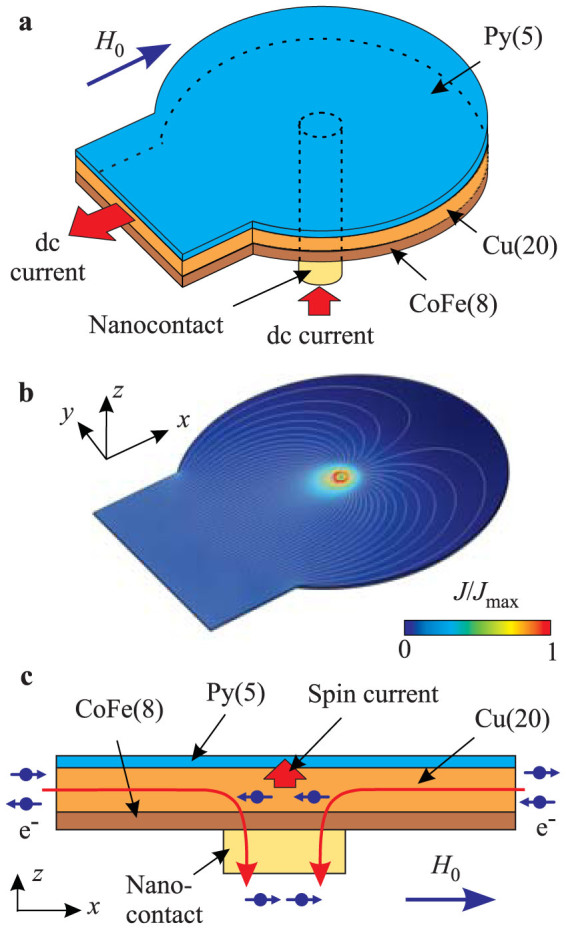
Experimental configuration. (a) Schematic of the experiment. (b) Calculated distribution of the charge current in the plane of the Cu layer. (c) Schematic illustration of the device operation principle. Arrows show the magnetic moments carried by the electrons.

Figure 1a shows the layout of the test devices. They are comprised by a multilayer Co_70_Fe_30_(8)Cu(20)Py(5) (Py = Permalloy = Ni_80_Fe_20_) disk with a diameter of 3 μm with a circular 60-nm diameter Au nanocontact in the center. A 2-μm wide strip attached to the side of the disk connects it to the external device contact. An electric current applied between the device electrodes flows from the nanocontact locally through the CoFe(8) polarizer. Since the resistivity of Cu is one order of magnitude smaller than that of Py, the current drains almost entirely through the Cu(20) spacer, while its flow through the Py(5) layer is negligible. Figure 1b shows the results of the calculation supporting this qualitative picture. In spite of the somewhat asymmetric electrode layout, the calculated distribution of current is almost radially symmetric in the vicinity of the point contact. By symmetry, this results in a negligible Oersted field produced by the current in the Py layer. Calculations of the current distribution through the vertical section of the multilayer show that 92% of the outflowing current is concentrated in the Cu layer. The contribution of the Py layer does not exceed 3%, which is significantly smaller than in the spin-Hall nano-oscillators involving high-resistivity spin Hall materials, where about 20% of the total current is typically shunted through the magnetic layer^28^. Since the driving current in our device flows predominantly through the low-resistivity layers, the Joule heating effects are minimized. In particular, heat flow simulations show that the increase of temperature in the active device region does not exceed 10 K within the range of currents used in our experiment.

The operation of the device relies on the spin accumulation at the Cu/CoFe interface above the nanocontact (Fig. 1c), caused by the spin-dependent resistivity of the CoFe layer and its interfaces^29^. The spin accumulation produces a spin current flowing into the Py layer due to the spin diffusion, exerting spin-transfer torque (STT) on its magnetization. The magnetizations of both CoFe and Py layers are aligned by the saturating static in-plane field *H*_0_. As seen from Fig. 1c, for positive driving electric currents as defined in Fig. 1a, the magnetic moment carried by the spin current is antiparallel to the magnetization of the Py layer. The resulting STT enhances fluctuations of the Py magnetization, which can be described as effective negative dynamic damping.

### Experimental data

The effect of STT exerted by the spin current on the magnetization of the Py layer was studied by micro-focus Brillouin light scattering (BLS) spectroscopy^30^ with the probing laser light focused into a diffraction-limited spot, yielding a signal proportional to the local intensity of the dynamic magnetization. This technique enables spectroscopic measurements of the magnetization dynamics with high sensitivity, allowing one to detect magnetic oscillations excited by thermal fluctuations.

**Figure 2:**
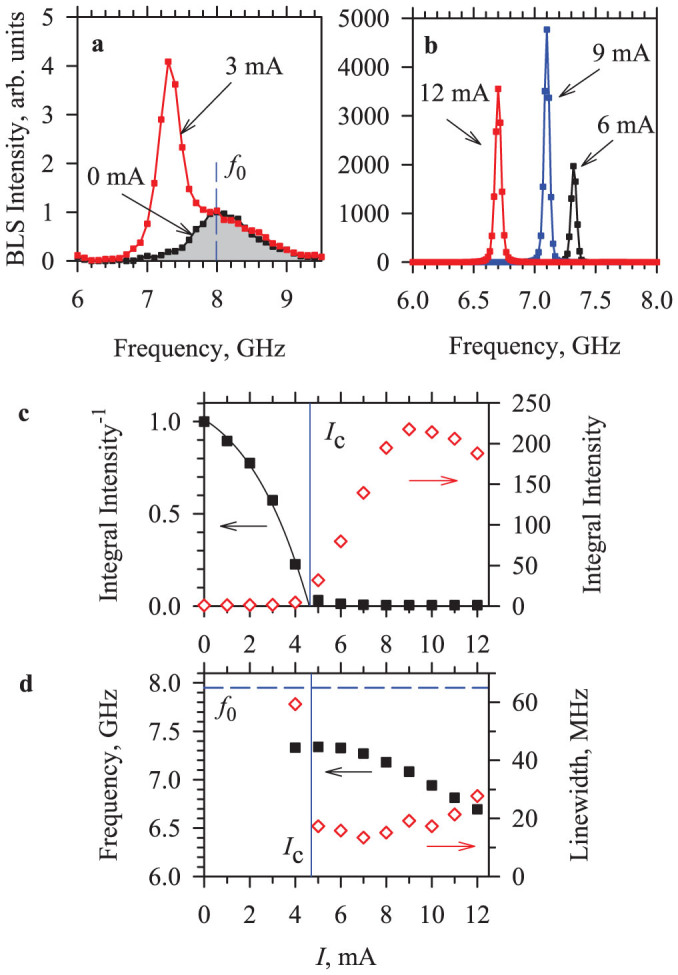
Spectroscopic characterization. (a) and (b) BLS spectra of magnetization oscillations in the test device recorded at the labeled current values, *f*_0_ marks the FMR frequency. (c) Integral BLS intensity (diamonds) and its inverse value (squares) *vs* current. The dependencies are normalized by the value at *I* = 0. *I*_C_ marks the threshold current for the onset of auto-oscillations. Curve is a guide for the eye. (d) Center frequency (squares) and the linewidth (diamonds) of the auto-oscillations *vs* current, at *H*_0_ = 750 Oe.

Figure 2a shows the BLS spectra of thermally excited magnetization dynamics in the Py layer recorded at *H*_0_ = 750 Oe. In the absence of the driving current (*I* = 0), the spectrum is broad and shows a maximum intensity at the frequency *f*_0_ corresponding to the frequency of the uniform ferromagnetic resonance (FMR) in the Py film. When a small positive current *I* = 3 mA is applied, the thermal magnetization fluctuations are enhanced, similar to the effects of spin current generated by the SHE^3^,^11^. A narrow peak whose intensity exceeds the thermal fluctuation background by several orders of magnitude emerges above a certain critical current *I*_c_ (Fig. 2b), marking the onset of auto-oscillations in the system.

The enhancement of the magnetization fluctuations and the onset of auto-oscillations were observed only at *I* > 0, while the fluctuations were increasingly suppressed with increasing magnitude of *I* < 0. The observed asymmetry of the current-induced effects is consistent with the expected effects of STT produced by the spin current on the dynamic damping and the fluctuations of the magnetization^3^. In contrast to the SHE-based systems^3^, the observed effects do not depend on the in-plane direction of the static magnetic field *H*_0_, since the magnetizations of both Py and CoFe layers are aligned with the field, always resulting in destabilizing STT at *I* > 0.

To characterize the processes resulting in the onset of auto-oscillations, in Fig. 2c we plot the dependencies of the integral BLS intensity and its inverse value on the driving current *I*. The integral intensity slowly grows at small *I*, abruptly increases at *I >* 4 mA, and then saturates at *I* > 10 mA. Extrapolation of the dependence of inverse intensity on current at *I* < 4 mA yielded an intercept value of *I*_C_ ≈ 4.7 mA. Above *I*_C_, the destabilizing torque exerted by the spin current completely compensates the magnetic damping and the system transitions to the auto-oscillation regime^3^,^11^,^31^. The onset of auto oscillations is also manifested by an abrupt drop of the detected spectral peak linewidth (diamonds in Fig. 2d) from 60 MHz at *I* = 4 mA to 17 MHz at *I* = 5 mA. At *I* < 10 mA, the linewidth remains in the range of 13 to 20 MHz. At larger currents, the spectral peak somewhat broadens and nonlinear saturation of the auto-oscillation amplitude is observed. We note that the linewidth was calculated from the BLS spectra as 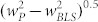, where *w_P_* is the full width at half maximum of the detected spectral peak and *w_BLS_* = 46.5 MHz is the full width at half maximum of the spectral transmission function of the BLS setup. The obtained value represents an upper estimate of the actual oscillation linewidth.

The frequency of the auto-oscillation peak (squares in Fig. 2d) exhibits a monotonic decrease with increasing *I* over the entire studied auto-oscillation range, consistent with the expected nonlinear frequency shift typical for in-plane magnetized films^32^. We note that the frequency of auto-oscillation is always smaller than *f*_0_ (dashed line in Fig. 2d), and is below the spectrum of propagating spin waves in the Py film. Therefore, propagating spin waves cannot be radiated by the oscillating mode into the surrounding Py film, and the oscillation is instead localized in the region of the nanocontact.

**Figure 3:**
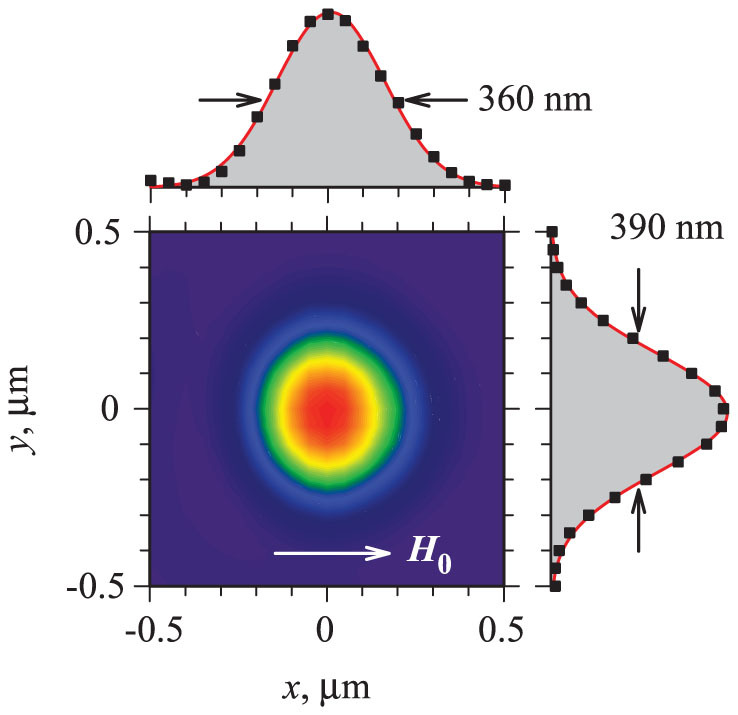
Spatial characteristics. Normalized color-coded spatial map of the measured BLS intensity proportional to the local intensity of magnetization oscillations, and two orthogonal sections through the center of the auto-oscillation area. Symbols are experimental data, and filled areas under solid curves are the results of fitting by a Gaussian function. The data were recorded at *I* = 8 mA and *H*_0_ = 750 Oe.

To further analyze the nature of the auto-oscillation mode, we recorded spatial maps of the dynamic magnetization, by rastering the probing laser spot over a 1 μm by 1 μm area around the nanocontact with the step size of 50 nm. A representative map acquired at *I* = 8 mA is shown in Fig. 3. The magnetization oscillations caused by the spin current are localized in the area above the nanocontact, in agreement with our analysis of the spectral characteristics. To determine the actual lateral dimensions of the localization area, one should take into account the broadening of the measured spatial profile due to the distribution of intensity in the diffraction-limited probing light spot whose calibrated diameter is 240–260 nm (Ref. 11). By deconvolving the recorded spatial profile shown in Fig. 3, we estimate that the actual size of the localization area is about 300 nm. This value is significantly larger than the diameter of the nanocontact (60 nm). We can infer that the localization area is likely determined by the lateral spin diffusion in the Cu layer. The values for the room-temperature spin-diffusion length in Cu reported in the literature range from 100 to 400 nm (Refs. 22, 23, 33), depending on the thickness, crystallinity, impurities, and properties of the interfaces. Even for the spin-diffusion length of 100 nm, the region of large spin accumulation (the region of the Py layer subjected to a large STT) significantly exceeds the size of the point contact.

**Figure 4:**
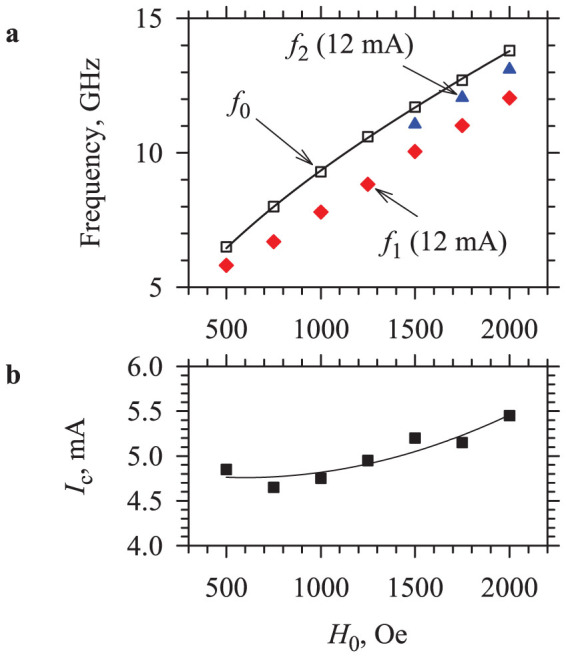
Field dependences. (a) Frequency of the fundamental (diamonds) and the secondary (triangles) auto-oscillation modes at *I* = 12 mA and the frequency of the ferromagnetic resonance in the Py film (squares) *vs* static field. Solid curve is the result of the fitting based on the Kittel formula. (b) Field dependence of the auto-oscillation onset current. Curve is a guide for the eye.

Behaviours similar to those described above were observed over a wide range of the static field *H*_0_. As shown in Fig. 4a, the auto-oscillation frequency (diamonds) is always below the FMR frequency *f*_0_ (squares). As the field is increased from 500 Oe to 2 kOe, the frequency monotonically increases by more than a factor of two from 5.8 GHz to 12.4 GHz. We note that the variation of the static field has a minimal effect on the onset current (Fig. 4b), which is promising for the applications requiring wide-range frequency tunability.

An additional peak appeared in the auto-oscillation spectrum at *H*_0_ > 1500 Oe, at a frequency higher than that of the primary peak, but still below the spectrum of the propagating modes (triangles in Fig. 4a). This mode becomes prominent only at large currents, where the amplitude of the primary mode exhibits saturation (Fig. 2c). We will show below that this secondary mode provides important information about the mechanisms of auto-oscillation.

## Discussion

Previous studies of in-plane magnetized nano-oscillators driven by the spin-polarized currents or by SHE have been successfully interpreted in terms of the nonlinear dynamical self-localization mechanism^28^,^32^. However, the oscillation region size of 300 nm observed in our experiment is significantly larger than that expected for the self-localized oscillation^11^,^28^. The self-localization mechanism is also expected to result in the abrupt onset of the auto-oscillation mode above the critical current, which is inconsistent with the observed gradual emergence of the oscillation peak (see Fig. 2a).

Here, we show that our observations can be entirely explained with a simple quasi-linear model of normal modes. We assume that the auto-oscillation modes in our device are linear normal modes of a circular region of the Py layer, where the effective static magnetization is reduced with respect to the rest of the layer due to the injection of spin current. This reduction is caused by the decrease of the static projection of the magnetization vector onto the precession axis due to the enhancement of magnetic fluctuations (incoherent spin waves with small wavelengths) by the spin current^3^,^31^, which is usually manifested by a nonlinear frequency shift of the dynamical magnetic modes and the nonlinear ferromagnetic resonance in the presence of the spin torque. Based on the data of Fig. 3, the diameter of the region with the reduced effective magnetization can be estimated as 300 nm. This region forms an effective potential well for the dynamical magnetization, with depth approximately proportional to the spin current strength. Localized dynamical magnetization states with discrete frequency values can then be formed in this effective well, in a direct analogy to the electronic quantum dots.

**Figure 5:**
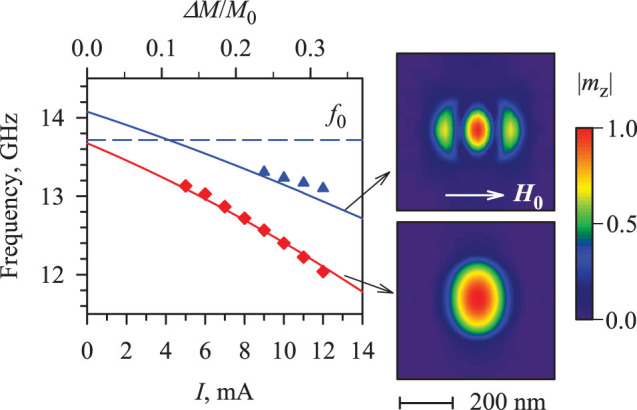
Micromagnetic simulations. Symbols: experimentally determined frequencies of the fundamental (diamonds) and the secondary (triangles) auto-oscillation modes *vs* current. Curves: simulated frequencies of the normal modes of a 300 nm diameter area of the Py film with the static magnetization reduced by the amount Δ*M*. Insets show the spatial profiles of the corresponding modes obtained from the micromagnetic simulations. All the shown data were obtained at *H*_0_ = 2000 Oe.

Figure 5 shows the results of the micromagnetic simulations of the normal modes performed with the software package MuMax3 (Ref. 34). Our system is modelled by a Py disc with the diameter of 1 μm and the thickness of 5 nm. The saturation magnetization 4π*M*_0_ = 10 kG, as found from the fit of the experimental data for the FMR frequency by the Kittel formula (Fig. 4a), is reduced in the central circular region with the diameter of 300 nm by the amount Δ*M/M*_0_ = *aI*, where *a* is the proportionality coefficient. The simulations were performed at *H*_0_ = 2000 Oe, when two auto-oscillation modes were clearly observed in the experiment.

As seen from Fig. 5, the experimentally determined frequencies of the auto-oscillation modes (symbols) are in excellent agreement with the calculated frequencies of the linear spin-wave modes localized in the potential well (lines). We emphasize that the only fitting parameter used in the simulation is the proportionality coefficient *a*, providing an accurate description of the frequencies of both modes and their dependencies on current. We also note that the value *a* = 0.025 mA^−1^ obtained from the fit is in a reasonable agreement with the experimental studies of the effects of spin current on magnetization^3^.

In addition to providing insight into the oscillation mechanism, simulations allow one to visualize the auto-oscillation modes on the spatial scale below the resolution of BLS. Insets in Fig. 5 show that the two auto-oscillation modes exhibit very different spatial characteristics: the fundamental mode has no nodes, while the secondary mode exhibits two nodal lines in the direction parallel to the static field. We also note that the profile of the fundamental mode is elongated in the direction perpendicular to the static field, in agreement with the experimental data of Fig. 3. We emphasize that our quasi-linear model does not take into account the subtle details of the spin diffusion and of the spin transfer torque that must be included in the thorough theoretical analysis of the studied system. Nevertheless, our model explains the observed spectral characteristics and provides an accurate quantitative description for the auto-oscillation frequencies.

Finally, we note that the onset of coherent auto-oscillations in our devices, as well as in other devices driven by the spin-transfer torque, can be qualitatively interpreted as the Bose-Einstein condensation of magnons^35^. However, in contrast to the experiments on the Bose-Einstein condensation of magnons in the Yttrium Iron garnet (YIG) films^36^, where a weak spin-lattice interaction and a long lifetime of magnons result in a quasi-equilibrium distribution of the excited magnon system, the thermodynamic description may not be directly applicable to the driven magnetic systems based on the metallic ferromagnets^37^.

In conclusion, we have demonstrated magnetic nano-oscillators based on the nonlocal spin injection. We showed that they exhibit a number of unique features such as negligible flow of the electrical current through the active ferromagnetic layer, negligible Oersted field of the driving current, wide-range frequency tunability, and narrow oscillation linewidth. Moreover, we find that the oscillation area in the studied devices is not limited by the self-localization effects and is relatively large, resulting in reduced thermal fluctuation effects. Efficient generation of a large microwave power by these oscillators can be also achieved by a straightforward addition of a tunneling magnetoresistance contact on top of the active magnetic layer. Additionally, thanks to the planar geometry, the proposed devices are promising for the implementation of local spin-wave sources for nanomagnonic applications^38^.

## Methods

### Sample fabrication

First, a Cu(30)Au(35) electrode is deposited by high-vacuum sputtering (all thicknesses are in nm). A circular 60 nm diameter Al(50) mask is fabricated by e-beam lithography and evaporation. The Au(35) layer is then removed from the electrode by Ar ion milling and an insulating Si(30) layer is deposited on top, followed by Ar ion milling almost parallel to the surface to remove Si from the Al mask. The latter is subsequently removed by etching in a dilute solution of HF, producing a flat Si-coated electrode surface (not shown in Fig. 1(a) for clarity) with a 60-nm diameter Au nanocontact. Finally, a Co_70_Fe_30_(8)Cu(20)Py(5) (Py = Permalloy = Ni_80_Fe_20_) multilayer disk with a diameter of 3 μm is fabricated on top of the nanocontact.

### Calculations of the current distribution

Calculations were performed using a three-dimensional finite-element numerical model of the sample. The three-dimensional problem based on the Maxwell equations was numerically solved using COMSOL Multiphysics® engineering simulation software (www.comsol.com). The computational domain was discretized by using a tetrahedral mesh with the smallest element size of 0.025 nm in the area of the point contact. Standard values of 6.4 × 10^6^, 6.0 × 10^7^, and 8.3 × 10^6^ S/m were used for the conductivities of Py, Cu, and CoFe, respectively.

### BLS measurements

Micro-focus BLS measurements were performed at room temperature by focusing light produced by a continuous-wave single-frequency laser operating at the wavelength of 532 nm into a diffraction-limited spot. The power of the probing light at the input of the focusing optics was 0.1 mW. The light inelastically scattered from the magnetization oscillations was analyzed by a six-pass Fabry-Perot interferometer TFP-1 (JRS Scientific Instruments, Switzerland) to obtain information about the BLS intensity proportional to the square of the amplitude of the dynamic magnetization at the location of the probing spot. By rastering the probing spot over the surface of the sample using a closed-loop piezo-scanner, two-dimensional maps of the spin-wave intensity were recorded with a resolution of 250 nm. The positioning system was stabilized by custom-designed active feedback, providing long-term spatial stability better than 50 nm.

### Micromagnetic simulations

The simulations were performed by using the software package MuMax3 available at http://mumax.github.io/. The computational domain was discretized into 5 × 5 × 5 nm^3^ cells. Standard Gilbert damping constant of 0.008 and the exchange stiffness of 1.3 × 10^−11^ J/m were used. The frequencies of the normal modes were calculated by analyzing the spectrum of the response of the dynamic magnetization to a short pulse of the magnetic field component perpendicular to the plane of Py layer. The temporal width of the pulse was 100 ps, and its amplitude was 0.01 Oe. The spatial profiles of the modes were reconstructed by plotting the two-dimensional spatial distribution of the absolute value of the Fourier component corresponding to the frequencies of the modes.

## Author Contributions

V.E.D. performed measurements and data analysis, S.U. suggested the idea of the experiment and fabricated the samples, A.Z. fabricated samples and performed their characterization, A.V.S. and A.N.S. performed modelling and the theoretical treatment of the experimental results, and S.O.D. formulated the experimental approach and performed the general supervision of the study. All authors co-wrote the manuscript.
